# The role of extracellular matrix proteins in gastric cancer development via epithelial-mesenchymal transition 

**Published:** 2020

**Authors:** Shima Abed Kahnamouei, Kaveh Baghaei, Parviz Pakzad, Mehrdad Hashemi, Mohammad Reza Zali

**Affiliations:** 1 *Department of Biology, Faculty of Biological Sciences, North Tehran Branch, Islamic Azad University, Tehran, Iran*; 2 *Basic and Molecular Epidemiology of Gastrointestinal Disorders Research Center, Research Institute for Gastroenterology and Liver Disease, Shahid Beheshti University of Medical Science, Tehran, Iran*; 3 *Department of Microbiology, Faculty of Basic Sciences, North Tehran Branch, Islamic Azad University, Tehran, Iran*; 4 *Department of Genetics, Faculty of Advanced Science and Technology, Tehran Medical Sciences, Islamic Azad University, Tehran, Iran*; 5 *Gastroenterology and Liver Diseases Research Center, Research Institute for Gastroenterology and Liver Diseases, Shahid Beheshti University of Medical Sciences, Tehran, Iran*

**Keywords:** Gastric cancer (GC), Epithelial mesenchymal transition (EMT), TGF- β cytokine, THBS2, OSMR and CHI3L1

## Abstract

**Aim:**

To acquire a deeper perception of EMT, we evaluated the expression of some candidate extra cellular matrix (ECM) proteins including THBS2, OSMR and CHI3L1 which were collected from RNA-seq bioinformatic analyses.

**Background:**

Gastric cancer (GC) is a major incident gastrointestinal cancer with a high rate of mortality. Metastasis is a challenging issue in gastric cancer treatment. Epithelial mesenchymal transition (EMT) of cancer cells is a complicated process controlled by different cells and molecular pathways regarded as an important step at the onset of metastasis.

**Methods:**

AGS gastric cancer cell line was cultured and treated by TGF-β. EMT induction was verified by measuring the expression of E-cadherin, Snail, β-catenin and Vimentin genes by real time PCR. Then, following our previous study, we evaluated the expression of THBS2, OSMR and CHI3L1 genes in EMT induced cells by real time PCR.

**Results:**

Downregulation of E-cadherin and upregulation of Snail, β-catenin and Vimentin genes were verified in AGS treated cells in comparison with none-treated cells (P-value = 0.0355, P-value = 0.007, P-value = 0.0059, P-value = 0.0206 respectively). Also, upregulation of THBS2, OSMR and CHI3L1 were validated in these cells after EMT induction (P-value = 0.0147, P-value = 0.05, P-value = 0.05 respectively).

**Conclusion:**

Our morphological and molecular results validated EMT induction by TGF- β cytokine in AGS gastric cancer cell line. Furthermore, significant upregulation of candidate genes including THBS2, OSMR and CHI3L1 verified the role of these proteins in gastric cancer invasiveness. However, further studies are needed for the validation of prognostic value of these markers.

## Introduction

 According to 2018 statistical reports, gastric cancer (GC) is known as the fifth incident cancer and the third cause of cancer death among men and women (1). Most cases are adenocarcinoma and clinically classified into two subgroups including intestinal and diffuse type (2). Metastasis and invasiveness of GC can justify high mortality more strongly than the cancer itself. Metastasis is the multi-step process including intravasation of primary tumor cells to blood vessels, circulation of these cells in blood, extravasation from blood vessels to tissues and colonization by making secondary tumors as a final destination (3). 

Epithelial mesenchymal transition (EMT) is a complicated process which is responsible in initiating and processing cancer metastasis by transforming epithelial cells to mesenchymal like cells with migratory phenotypes(3,4). Tumor microenvironment has the obvious impact on EMT(5). Altering cell-cell and cell-extra cellular matrix (ECM) interactions caused by cytokines and chemokines through signaling pathways provide a proper microenvironment by expressing EMT transcription factors like Snail, Twist, Zeb and Slug. These downstream transcription factors affect the expression of cytoskeleton proteins such as E-cadherin which has an important role in the structure of epithelial cells. Consequentially, decreasing of E-cadherin replacing by N-cadherin accompanying with other EMT associated markers such as Vimentin, Fibronectin and β-catenin transform epithelial cancer cells to mesenchymal like behaved cells with invasive phenotype(6,7).

**Table1:** The sequence of primers for E-cadherin, Snail, β-catenin, Vimentin and GAPDH genes

Gene name	Primer sequence 5'→ 3'
E-cadherin F E-cadherin R	TGCTCTTGCTGTTTCTTCGG CTTCTCCGCCTCCTTCTTC
Snail F Snail R	CACTATGCCGCGCTCTTTC TGCTGGAAGGTAAACTCTGGAT
β -catenin F β -catenin R	GGGTAGGGTAAATCAGTAAGAGGT GCATCGTATCACAGCAGGTT
Vimentin F Vimentin R	CCAGGCAAAGCAGGAGTC CGAAGGTGACGAGCCATT
GAPDH F GAPDH R	TGAAGGTCGGAGTCAACGGATTTGGT CATGTGGGCCATGAGGTCCACCAC

**Table 2:** The sequence of primers for THBS2, OSMR and CHI3L1 genes

Gene name	Primer sequence 5'→ 3'
THBS2 F THBS2 R	ACTTCAGGGGTTTGCTTCAG GTGTTCTCACTGATGGCGTTG
OSMR F OSMR R	CGTTTACCATTGACTCCTGT AATTCCCCACCCAGATGAC
CHI3L1 F CHI3L1 R	CTCAAGAACAGGAACCCC TCCAGCCCATCAAAGCCAT

ACRG present a new molecular classification for Asian gastric cancer based on their genetic signature that divided this cancer into four subgroups including: 1. Gastric cancer with MSI; 2. gastric cancer with MSS and EMT phenotype; 3. Gastric cancer with P53 alterations; and 4. Gastric cancer without P53 alterations. In this classification, MSS/EMT group showed extremely low survival rates, poor prognosis and high rate of further recurrence in comparison with others(8).

In this study, we tried to induce EMT in AGS gastric cancer cell line by TGF- β cytokine which is known as a powerful EMT inducer through SMAD dependent or independent molecular pathways(9). TGF- β could play two completely different roles in normal and tumor cells. In normal or premalignant cells, TGF-β is a major tumor suppressor which could arrest cell cycle in early phases or induce apoptosis in early stages of malignancies. However, in late stages of tumorigenesis, TGF- β shows its oncogenic role by the induction of EMT phenotype in cancer cells(10). After TGF- β treatment, we confirmed EMT induction by evaluating the expression of EMT-related markers. 

Following that, we evaluated the expression of THBS2, OSMR and CHI3L1 in EMT-induced AGS cells as a final purpose. 

## Methods

**Bioinformatic analysis**

In our previous study, we performed RNA-seq bioinformatic analysis through DESeq2 package (R software) to find differentially expressed genes in GC. Then, pathway enrichment analysis was performed to help select candidate genes(11).

**Cell culture**

In vitro experiment was carried out using AGS (Human gastric cancer) cell line collected from Research Institute of Gastroenterology and Liver Diseases (Shahid Beheshti university of medical science, Tehran, Iran) and were cultured in RPMI (Roswell Park Memorial Institute) medium (Gibco-Invitrogen, USA) completed with 10% FBS, 1% pen-strep antibiotic, 1% non-essential amino acids, and 1% L-glutamine (Gibco-Invitrogen, USA) in 75 T flask incubated at 37 °C with 5% CO2 for 48-72 hours to make proper confluency. 

**EMT induction**

When cells reached proper confluency, 50000 cells were seeded in two 25T flasks in RPMI medium. For EMT induction, we starved one of our flasks with 0.5% FBS and treated cells with 10 ng/ml of TGF- β for 48 h simultaneously. The other flask was cultured keeping previous conditions with 10% FBS as the control group.

**RNA extraction and cDNA synthesis**

RNA was extracted by QIAGEN All Prep DNA/RNA/miRNA universal kit (Cat. No.: 80224) step by step according to the protocols. Then, the quality of RNAs were checked by measuring the optical density with the Nanodrop (Thermo Fisher Scientific, USA). The optimum ratio of the absorbance at 260 /280 nm should be from 1.8 to 2.0. Finally, cDNA was synthetized by Thermo fisher Revert Aid First Strand cDNA Synthesis Kit using Random Hexamer primers (Cat. No.: k1691).

**Verification of EMT induction by real time PCR** Primers were designed by Primer 3 online software for EMT associated markers including E-cadherin, Snail, β-catenin and Vimentin genes (Table.1). Designed primers were checked by Gene Runner tool (version 6.0.04) and were blasted at NCBI. 

**Figure 1:**
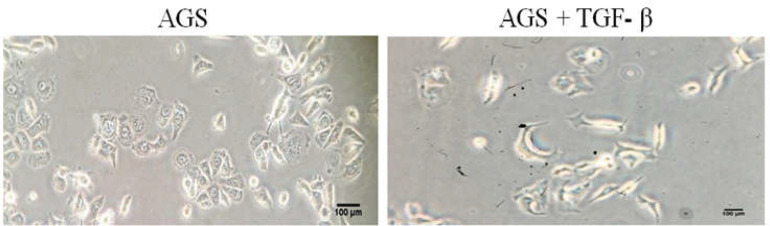
Morphological changes in AGS cell line after TGF- β treatment (200x)

**Figure 2:**
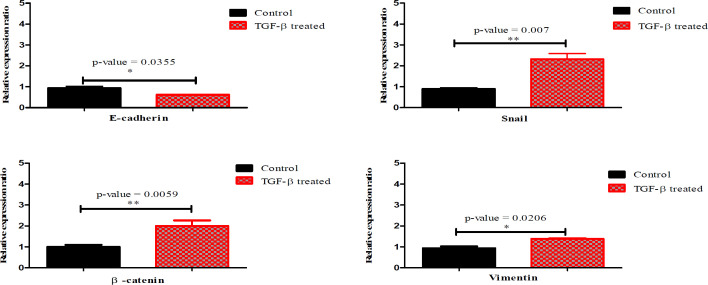
Validation of EMT induction by measuring mRNA level of EMT associated markers between treated and non-treated AGS cells. The genes were normalized by GAPDH as a housekeeping gene (P-value < 0.05). The data is presented as SEM of three times experiments (**P<0.01, ***P<0.001 and ****P<0.0001)

**Figure 3:**
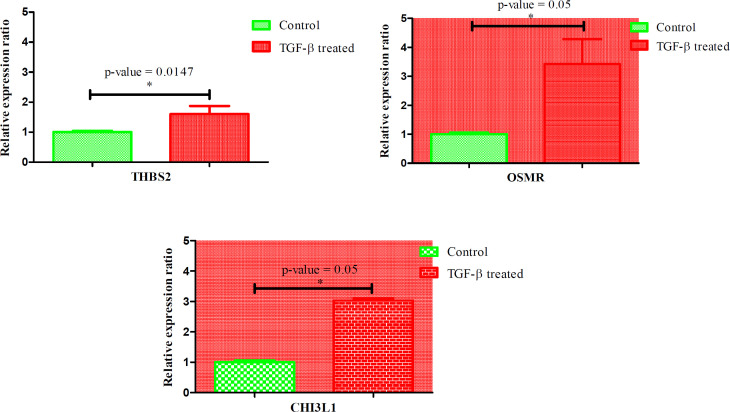
Validation of differentially expressed mRNA of THBS2, OSMR and CHI3L1 genes between EMT-induced cells and control (non-treated) cells. All genes were normalized by GAPDH. Upregulation of these three genes were verified significantly (P-value < 0.05). The data is presented as SEM of three times experiments (**P<0.01, ***P<0.001 and ****P<0.0001)

Real time PCR was carried out in a total amount of 25 μl using Amplicon SYBR Green without ROX (Cat. No.; A323402) in order to measure the expression of targeted mRNAs in treated and non-treated AGS cells. GAPDH (Glyceraldehyde-3-phosphate dehydrogenase) gene was used as an internal control and the non-template samples were employed as a negative control in each reaction. PCR was carried out on a Rotor-Gene Q real-time PCR machine. Initial denaturation was performed at 95°C for 15 minutes in order to activate the Hot start Taq DNA polymerase. Then, for 40 cycles, ampliﬁcation (at 95°C for the 30 seconds) and annealing (at appropriate temperature for each gene for the 60 seconds) were performed in a two-step program. 

**Validation of THBS2, OSMR and CHI3L1 genes by real time PCR in EMT induced cells**

After verification of EMT induction, expression of THBS2, OSMR and CHI3L1 genes were measured in TGF- β treated and non-treated cells by real time PCR. The procedure of primer design (Table.2) and the condition of real time PCR were the same as the aforementioned steps.

**Statistical analyses**

Real time PCR analysis was performed by REST 2009 (version 2.0.13) and Prism5 software. All data are presented as the mean ± SEM and expression ratio were analyzed using one tailed, unpaired t-test (P value < 0.05). 

## Results

By considering biological function of upregulated and downregulated genes in GC, we selected three genes from top ten with the highest expression including THBS2, OSMR and CHI3L1 which have a major role in ECM remodeling.

In our Microscopic findings, obvious morphology changes in AGS cells were observed by inverted microscope (Olympus, Japan). 

We observed colonies of cells with their typic epithelial forms in non-treated flask as our control group and transformed cells with spindle like shape in TGF- β treated flask (Figure 1).

To consider the expression level of ECM changes, real time PCR technique was applied. Besides, downregulation of E-cadherin gene and upregulation of Snail, β-catenin and Vimentin genes were confirmed in treated cells compared with non-treated cells (P-value < 0.05) (Figure 2). 

In the next phase of our experiment, results of real time PCR showed significant upregulation of THBS2, OSMR and CHI3L1 genes in EMT-induced AGS cells compared with control cells (P value < 0.05) (Figure 3).

## Discussion

In spite of different molecular and pathological classifications, poor prognosis and low survival rate of gastric cancer is still challenging and many cases are diagnosed in late metastatic stages (8,12). Due to the importance of metastasis and molecular heterogenicity of GC, we aimed to acquire better perception of tumor microenvironment which has an obvious impact on EMT initiation. 

In our previous study, we carried out RNA-seq bioinformatic reanalysis of Asian published raw dataset and we reached differentially expressed genes in GC of Asian patients. Then, we selected and validated three genes including THBS2, OSMR and CHI3L1 in Iranian gastric cancer patients. These genes were selected from top ten upregulated ones which have roles in ECM remodeling(11). 

In this experiment, we validated these three genes in EMT invitro model of AGS cell line. THBS2, OSMR and CHI3L1were overexpressed as the results of our previous study (Figure.3). 

In recent decades, the role of EMT was reported in metastasis and invasiveness of many cancers (13,14). In this study, THBS2 is one of the targeted genes which is considered to have a role in GC invasiveness. Consistent with our results, THBS2 was regarded as a potential prognostic biomarker for colorectal cancer (CRC). According to this study, meta-analysis of GEO and TCGA datasets showed the relation between THBS2 expression level with tumor metastasis and prognosis. Also, the mentioned study showed the correlation between THBS2 and EMT markers like Snail, MMP9 and Vimentin. Furthermore, pathway enrichment analysis of this study demonstrated the role of this glycoprotein in ECM interactions, focal adhesions and activation of TGF- β cytokine. All these paths are involved in the activation of EMT process (15). In line with our study, in another study by Zhuo et al., differentially expressed genes among 105 GC patients and controls were investigated. Forty-three genes were identified according to their microarray results. THBS2, COL1A2, and SPP1 genes, which have an important role in ECM interactions, were selected and verified by RT PCR, western blot and immunohistochemistry(16). 

OSMR was another targeted gene in our experiment which has a role in the activation of EMT and cancer progress(17). Like our findings, Zhenjia Yu et al. showed upregulation of OSMR gene in GC patients and gastric cancer cell lines. Accordingly,, a correlation between expression of OSMR gene and N-cadherin gene was verified in GC tissues compared with paired controls and knockdown of OSMR gene decreased invasion and migration of GC cells. Furthermore, this study suggested that SP1 transcription factor induces the expression of this gene by attachment to its promoter (18).

The last targeted gene in this study was CHI3L1 which was reported as a serum biomarker in many cancers(19). Qiu et al. reported the role of this gene in hepatocellular cancer promotion and invasion, both in vitro and in vivo. According to their RNA-seq results, upregulation of this gene could affect the expression of other genes which were involved in ECM adherence. In addition, their western blot analysis showed that this glycoprotein could activate TGF- β cytokine through SMAD dependent pathway(20). Consistent with our results, Geng et al. reported CHI3L1 as a novel biomarker in GC prognosis with more advance phenotypes. They showed that CHI3L1- CD44 could activate upregulation of β-catenin which has an obvious role in EMT process through ERK/AKT signaling pathway (21).

Our findings demonstrated a correlation between ECM genes and EMT during gastric cancer diseases. Upregulation of THBS2, OSMR and CHI3L1 was verified in EMT-induced gastric cancer cell line. In line with our results and other researches, all these three genes have roles in EMT process, mostly by modifying ECM structure.
